# Radioiodine thyroid remnant ablation in patients with differentiated thyroid carcinoma (DTC): prospective comparison of long-term outcomes of treatment with 30, 60 and 100 mCi

**DOI:** 10.1186/1756-6614-3-9

**Published:** 2010-11-01

**Authors:** Aleksandra Kukulska, Jolanta Krajewska, Marzena Gawkowska-Suwińska, Zbigniew Puch, Ewa Paliczka-Cieslik, Jozef Roskosz, Daria Handkiewicz-Junak, Michał Jarzab, Elzbieta Gubała, Barbara Jarzab

**Affiliations:** 1 Department of Nuclear Medicine and Endocrine Oncology, M. Skłodowska-Curie Memorial Cancer Center and Institute of Oncology, Gliwice Branch, Gliwice, Poland

## Abstract

**Background:**

The aim of this study is to compare the effectiveness of ^131^I therapy between three groups of DTC patients who received 30, 60 or 100 mCi for thyroid remnant ablation after total thyroidectomy and were postoperatively judged with low risk of cancer recurrence.

**Methods:**

The project was designed as a two-stage, prospective randomized clinical trial. In 1998-2001 in a randomized prospective study the early comparison of treatment with 30 mCi vs 60 mCi suggested the lower ^131^I activity to be less effective, whereas in 2003-2005 the comparison between 60 vs 100 mCi showed no significant differences. The present study comprises the long-term assessment of the disease course in 3 study groups.

**Results:**

A group of 309 DTC patients (285 women and 24 men) with no clinical, histopathological, sonographical or biochemical signs of persistent disease were included after total thyroidectomy and appropriate extent of neck lymph node dissection (265 with papillary and 44 with follicular thyroid cancer). For radioiodine thyroid remnant ablation, 30 mCi of ^131^I was applied in 86 patients, whereas 60 mCi in 128 and 100 mCi in 95 patients. The median follow-up was 10 years (2-12) for subjects treated with 30 mCi and 60 mCi and 6 years (2-6) for patients treated with 100 mCi of ^131^I. In the first evaluation, published previously, we observed that because of incomplete thyroid remnant ablation, the second ^131^I treatment was necessary in 10% patients, without difference between groups treated with 60 and 100 mCi and in 22% patients treated with 30 mCi. All patients entered full remission. To evaluate the long-term outcome of the adjuvant ^131^I treatment, the course of the follow-up and the most recent disease status were assessed by sonography, radiological examinations and serum Tg estimation (on LT4-suppressive treatment). Within the whole observation period local relapse was stated in 2 (2.4%), 4 (3%) and 3 (3%) patients treated with ^131^I activities of 30 mCi, 60 mCi and 100 mCi respectively and serum Tg concentration on LT4-suppressive treatment was low, without differences between groups.

**Conclusions:**

No significant differences in the 5 years efficacy of thyroid remnant radioiodine ablation using 30, 60 and 100 mCi were observed in low-risk DTC patients operated by total thyroidectomy and neck lymph node dissection. However, patients treated initially with 30 mCi, required second course of radioiodine in 22%, while this was necessary only in 13,3% and 11,2% of patients treated with 60 mCi and 100 mCi respectively.

## Introduction

Postoperative complementary radioiodine (^131^I) therapy is the standard treatment for differentiated thyroid cancers (DTC). One aim of this therapy is to destroy thyroid remnant to remove their competition with cancer cells for secretion of thyroglobulin or ^131^I uptake. Thus, the name of thyroid remnant ablation is used. The second aim is to treat putative cancer micrometastases. Only the adjuvant ^131^I therapy enables the detection of functional micrometastases, which are visible during the post-therapeutic total body scan, but not on X-ray or CT images [1]. Therefore it is important to ensure, both for the healthy follicular cells and thyroid cancer cells to absorb appropriately high radiation doses. The absorbed dose depend, among others, on the ability of the cells to trap and cumulate ^131^I. The capability of thyroid cancer cells is much lower than of the healthy ones so the ^131^I activity sufficient for thyroid remnant ablation might not be sufficient to treat cancer micrometastases. That is why the evaluation of the effectiveness of the adjuvant ^131^I therapy must include the assessment of thyroid remnant ablation effectiveness as well as of micrometastases treatment. The latter can be done only by the long-term disease follow-up.

Usually, most DTC recurrences are diagnosed within first 5 years postoperatively. Adjuvant ^131^I treatment increases specificity of the further serum thyroglobulin follow-up, as in patients operated because of differentiated thyroid carcinoma but not treated by ^131^I the source of Tg might be both healthy thyroid cells and cancer cells. After successful thyroid remnant ablation thyroglobulin may be secreted by only cancer cells. Also the adjuvant therapy improves the sensitivity and specificity of the further DTC ^131^I-based diagnostic by abolishing the competition amongst healthy follicular cells and cancer cells to uptake the ^131^I.

For patients with differentiated thyroid cancer various therapeutic activities are used to carry out the adjuvant ^131^I treatment: from 30 to 200 mCi (1,1 - 7,4 GBq) [2-7]. The most favorable activity has not been established yet although a great deal of research has been performed. Early papers evaluating the efficiency of the adjuvant ^131^I treatment mainly focused on its thyroid ablation role and were rather observational. They did not prove any relation between therapeutic activity and ablation effectiveness. In 1996, Bal et al. carried out a prospective randomized clinical trial in which they evaluated the effectiveness of some activities. Then divided the patients into four groups which were administered respectively: 25-34 mCi, 35-64 mCi, 65-119 mCi and finally 120-200 mCi. They concluded that 50 mCi was the most effective activity and that an increase in activity did not bring about any therapeutic benefits. Mazaferri compared retrospectively the outcome in 1,004 patients with differentiated thyroid carcinoma, whose median follow-up time was on average 15 years, however, did not indicate the optimal dose of ^131^I necessary to achieve the best therapeutic effect. The prospective randomized study published in 2008 by Mäenpää et al. evaluated the effectiveness of the thyroid remnant ablation and long-term results of adjuvant ^131^I therapy and did not prove the treatment with 100 mCi being better than 30 mCi [6]. Thus, we decided to perform own prospective study. The aim of this study is to compare the effectiveness of adjuvant ^131^I therapy between three groups of DTC patients who were treated by total thyroidectomy and lymph neck dissection and evaluated postoperatively to be free from disease. They received respectively 30, 60 and 100 mCi of radioiodine for thyroid remnant ablation.

## Patients and methods

The project was designed as a two-stage, prospective randomized clinical trial.

The first part of the study was an early evaluation of the effectiveness of thyroid remnant ^131^I ablation treatment and was described in our previous papers [8-10]. In 1998-2001 210 low risk DTC patients, diagnosed with differentiated thyroid carcinoma, were randomly allocated to receive either 1100 MBq (30 mCi), or 2200 MBq (60 mCi) activity of ^131^I following total thyroidectomy and in most cases, central lymph node dissection [8,9]. Only patients without lymph node metastases, staged pT_1b_-T_3 _or T_x _and N_0 _or N_x _were included. The condition to show stimulated Tg level <30 ng/ml was fulfilled for all of them.

From 2003 to 2005 as the completion of the range of ^131^I activities analyzed, we compared the effectiveness of two ^131^I activities, 60 mCi and 100 mCi. For this goal, 224 patients staged pT_1b_-T_4 _N_0-1 _were included, if they were diagnosed disease-free following total thyroidectomy and central/lateral lymph node dissection, when necessary.

^131^I treatment was performed after thyroid hormone withdrawal. A successful thyroid remnant ablation was defined as the absence of thyroid bed uptake in ^131^I neck scan and the stimulated thyroglobulin (Tg) value < 10 ng/ml, as judged after 12 months post ^131^I therapy [10].

The second stage of the study, presented now, comprised the long-term assessment of the disease follow-up in the study groups. 309 DTC patients (285 women and 24 men) with no clinical signs of persistent disease were included. There were 267 subjects with papillary and 44 with follicular carcinoma. 86 patients were initially treated with 30 mCi (group A), 128 with 60 mCi (group B) and 95 with 100 mCi (group C) post thyroidectomy.

In group A there were only patients in pT_1b_-T_3 _N_0 _M_0 _stage, in other cohorts the wider range of patients in pT_1b_-T_4_, N_0_-N_1 _M_0 _were included. The distribution of patients in early stages of the disease, who constituted the majority, in all groups was similar (table 1).

**Table 1:** TNM classification of DTC patients

	T1b	T2	T3	T4	Tx		N0	N1	Nx	Total
Group A (30 mCi)	13 (15%)	42 (48%)	9 (10%)	0	20 (25%)	86 (100%)	46 (53%)	0	40 (47%)	86 (100%)

Group B (60 mCi)	37 (29%)	59 (46%)	11 (8%)	7 (5%)	14 (10%)	128 (100%)	74 (57%)	5 (4%)	49 (39%)	128 (100%)

Group C (100 mCi)	22 (23%)	50 (52%)	5 (4%)	7 (7%)	13 12(%)	95 (100%)	53 (55%)	13 (14%)	29 (31%)	95 (100%)

Total	72	151	25	14	47	309	173	18	118	309

The median follow-up was 10 years (range 2-11 years) for subjects treated with 30 mCi and 60 mCi and 6 years (range 2-6 years) for patients treated with 100 mCi of ^131^I. Only 5 patients in group A and 12 in group B and C were observed less than 6 years.

The most recent evaluation of the disease status and outcome were assessed by sonography, radiological examinations and serum Tg level (on LT4-suppressive treatment).

## Results

In the early evaluation, described in details elsewhere [8,9], there were 22% cases of incomplete thyroid remnant ablation after 30 mCi of ^131^I, requiring the second ^131^I therapy and 13,3% and 11,2% after 60 and 100 mCi respectively. The freedom from disease was confirmed by the low stimulated Tg in all patients during the evaluation done 6-12 months after the completion of adjuvant ^131^I therapy.

During the whole follow-up, the local relapse was stated in 2 (2.4%), 4 (3%) and 3 (3%) patients treated with ^131^I activities of 30 mCi, 60 mCi and 100 mCi respectively, without differences between groups.

If only patients in early stage of the disease were included (291 pT1-3,N0-x), local relapse was observed in 2 patients in group A and 3 and 2 in groups B and C respectively. Locoregional relapses were diagnosed after 8 and 3 years after surgery in group A, after 2, 4 and 9 years after surgery in group B and 1, 2, 3 years after surgery in group C, without any differences between groups.

During the whole follow-up we did not observe any distant recurrence in any of the patient.

The median last serum Tg level on LT4-suppressive treatment was low in all groups. Serum Tg concentration above the threshold of the method's sensitivity was observed in only one third of patients. Just a few subjects presented an increase serum level Tg without any localization of the cancer focus (table 2).

**Table 2:** Serum Tg level on LT4-suppressive treatment, as judged during the most recent examination in all patients.

Serum Tg level	GROUP A	GROUP B	GROUP C
No	**86**	**128**	**95**

**Median**	**0,1 ng/ml**	**0,17 ng/ml**	**0,17 ng/ml**

Higher than 0,17 ng/ml	21%	28%	29%

Maximal	1,1 ng/ml	6,4 ng/ml	8,15 ng/ml

Number of patients showing Tg>1 ng/ml	1	4*	4*

*In none of the patients the extended evaluation, including ^131^I scan, revealed disease foci.

Tg serum level evaluated on LT4-suppressive treatment analyzed in the group of patients with pT_1-3_,N_0-x _resembled data on lack of differences in Tg serum level in the whole group.

For some patients we performed a full diagnostic procedure during endogenous TSH stimulation, especially for those with slightly increased suppressed Tg level. For all of them stimulated Tg serum level was low and there were no significant differences between the groups. There were no localized disease cases either by ^131^I scan or by radiological examinations.

## Discussion

Postoperative ^131^I therapy is the standard treatment for differentiated thyroid cancers.

In the USA it is only used with patients in more advanced DTC stage [2,11]. In such cases the effectiveness of this method is undisputed. It has been proved that it decreases the risk of the recurrence and death due to DTC [11,12]. It is more commonly used in Europe although it is still questionable if it is necessary with the patients in early disease stage [13]. However, it is important to remember that it allows a better follow-up through the improvement of specificity of serum thyroglobulin measurement and sensitivity of the whole body scan. Thus, it is routinely used in many European countries and recommended by Polish guidelines in all DTC cases but papillary microcancer [14]. In our clinical practice we use routinely 100 mCi ^131^I for thyroid remnant ablation. However, being aware of the possibility to use lesser activities, we decided to perform a prospective randomized trial with long term outcome. We plan to extend our observation till 10 years follow up will be reached, however, an interim analysis has been planed after 5 years and this short communication present its result. In the previous, initial part of the study we found that the 30 mCi activity was less effective than 60 mCi as more patients needed second ^131^I treatment [9], but 60 mCi was as good as 100 mCi [10]. The later evaluation of the therapeutic effect, presented in this paper, did not show significant differences in the effectiveness of 30, 60 and 100 mCi activities. In our study the majority of patients were diagnosed with early stage of the disease, so the results are related to low risk DTC patients treated by radical surgery. However, one limitation is obvious: in the group treated initially by 30 mCi of ^131^I 22% of patients required second course of ^131^I for complete thyroid ablation. Thus, in fact, the cumulative ^131^I activity administered was larger in 22% of patients and exceeded 100 mCi. The same occurred in 13,3% and 11,2% of patients treated with 60 and 100 mCi respectively. This will be considered at the final evaluation, however, already now we have to bear in mind that the apparent equality of various activities of ^131^I has to be interpreted with caution. All for whom the initial treatment proved insufficient during the first assessment were treated again with a higher activity which might have influenced the good general outcome of radioiodine treatment in the group treated with lower activities.

## Conclusions

In the analyzed groups of patients no significant differences in 5 years efficacy of ^131^I thyroid remnant ablation were observed. However, to reach this goal, 22% of patients treated with 30 mCi required second course of radioiodine, while this was necessary only in 13,3% and 11,2% of patients treated with 60 and 100 mCi respectively.

## Competing interests

The authors declare that they have no competing interests.

## Authors' contributions

AK and JK conceived the research work, coordinated the data collection and prepared the manuscript; MGS reviewed patients data, ZP reviewed patients data, EPC reviewed patients data, JR reviewed patients data, DHJ reviewed patients records and performed the statistical analysis, MJ reviewed patients records and performed the statistical analysis, EG performed the statistical analysis, BJ exerted critical revision and supervision; The manuscript has been seen and approved by all authors
